# Pharmacogenetics and Age-Related Macular Degeneration

**DOI:** 10.1155/2011/252549

**Published:** 2011-10-20

**Authors:** Stephen G. Schwartz, Milam A. Brantley

**Affiliations:** ^1^Bascom Palmer Eye Institute, Miller School of Medicine, University of Miami, 311 9th Street North, No. 100, Naples, FL 34102, USA; ^2^Vanderbilt Eye Institute, Vanderbilt University Medical Center, 2311 Pierce Avenue, Nashville, TN 37232, USA

## Abstract

Pharmacogenetics seeks to explain interpatient variability in response to medications by investigating genotype-phenotype correlations. There is a small but growing body of data regarding the pharmacogenetics of both nonexudative and exudative age-related macular degeneration. Most reported data concern polymorphisms in the *complement factor H* and *age-related maculopathy susceptibility 2* genes. At this time, the data are not consistent and no definite conclusions may be drawn. As clinical trials data continue to accumulate, these relationships may become more apparent.

## 1. Introduction

Pharmacogenetics, an evolving research discipline within ophthalmology, investigates genotype-phenotype correlations in an attempt to explain interpatient variability in response to medications. While the earliest ophthalmic pharmacogenetic reports involved the treatment of open-angle glaucoma [1, 2], there is now a growing body of data concerning various treatments for age-related macular degeneration (AMD).

The combination of antioxidants and zinc studied by the Age-Related Eye Disease Study (AREDS) was reported to reduce disease progression and visual loss in certain patients with nonexudative AMD [3]. These supplements remain the only clinically proven treatment for nonexudative AMD. A variety of treatments have demonstrated efficacy in the treatment of choroidal neovascularization (CNV) secondary to exudative AMD, including photodynamic therapy (PDT) with verteporfin (Visudyne, Novartis, Basel, Switzerland) and the antivascular endothelial growth factor (VEGF) agents. Currently, there are three anti-VEGF agents in clinical use in the US: pegaptanib (Macugen, Eyetech, Palm Beach Gardens, Fla) [4], ranibizumab (Lucentis, Genentech, South San Francisco, Calif) [5, 6], and bevacizumab (Avastin, Genentech, South San Francisco, Calif) [7]. Despite the overall efficacy of these treatments, there remains a persistent and unexplained variability in treatment response with certain patients, especially those treated with anti-VEGF agents [8]. Intravitreal triamcinolone acetonide has been reported to show some efficacy as an adjunctive therapy in some patients with CNV, especially when combined with PDT [9] or bevacizumab [10]. Unfortunately, elevation of intraocular pressure is an important adverse event associated with this treatment [11].

Pharmacogenetics may help to explain some of this variability in treatment efficacy and toxicity.

## 2. Studied Genotypes

The complement system appears to play an important role in the pathogenesis of AMD [12]. Recent studies demonstrated that a single nucleotide polymorphism (SNP) in the complement factor H (*CFH*) gene is strongly linked with AMD [13–16]. As a primary regulator of the complement cascade, CFH plays an important role in innate immunity and inflammatory response. In these studies, individuals with one risk allele for this SNP (genotype TC) had a significantly increased risk of AMD (odds ratios (ORs) ranging from 2.5 to 4.6), and two risk alleles (genotype CC) conferred a correspondingly higher risk (ORs ranging from 3.3 to 7.4). Multiple reports have confirmed this association in different populations [17–21]. The influence of the complement pathway on AMD was further validated when polymorphisms in the complement factor B/C2 (*CFB*), C3, factor I (*FI*), and CFH-related proteins 1 and 3 were also shown to influence AMD susceptibility [22–27]. 

A second locus, encompassing the *ARMS2 *(*age-related maculopathy susceptibility 2*, also called *LOC387715*) and *HTRA1 *(*HtrA serine peptidase 1*) genes on chromosome 10q26, has also been consistently associated with AMD [28–31]. It has proven difficult to determine whether variants in *ARMS2 *or *HTRA1* are responsible for the association with AMD because they are in strong linkage disequilibrium and their effects are statistically indistinguishable. The function of the ARMS2 protein is unknown. There is some evidence that the *HTRA1 *polymorphism is functional and influences gene expression, but these data have been inconsistent, and this continues to be debated [32–38]. 

Polymorphisms in numerous other genes may exert smaller effects on AMD susceptibility. Two recent genome-wide association studies (GWAS) showed that the hepatic lipase (*LIPC*) and tissue inhibitor of metalloprotease 3 (*TIMP3*) genes may influence AMD risk [39, 40]. 

Apolipoprotein E levels, encoded by *APOE*, also have been associated with AMD [41]. Although *VEGF* (also known as *VEGFA*) has not been reported to be a major AMD susceptibility locus, polymorphisms within this gene have been associated with exudative AMD in some studies [42, 43]. Similarly, VEGF receptor 2, encoded by *kinase insert domain receptor* (*KDR*), may play a role in the development of CNV [44]. Plasma levels of C-reactive protein, encoded by *C-reactive protein *(*CRP*), have been associated with AMD [45, 46]. *Low-density lipoprotein receptor-related protein 5 *(*LRP5*) and *frizzled homolog 4 *(*FZD4*) have been associated with retinal vascularization but not specifically with AMD [47]. *Pigment epithelium-derived factor *(*PEDF*) polymorphisms have also been studied in AMD patients [48].

The glucocorticoid receptor gene (*GR*) has six well-studied polymorphisms: *ER22/23EK *[49], *N363S *[50], *Bcl*I [51], *N766N*, a substitution within intron 3, and a substitution within intron 4 [52]. None of these SNPs is reported to represent an AMD susceptibility locus, but several have been associated with altered sensitivity to glucocorticoids in nonophthalmic studies.

## 3. Pharmacogenomics of AREDS Vitamins

A subset of patients studied in the AREDS trials was evaluated for a pharmacogenetic response with respect to polymorphisms in *CFH* Y402H and *ARMS2*/*LOC387715 *A69S (Table 1). A total of 264 of 876 AREDS category 3 and 4 patients (30.1%) progressed to advanced AMD over five years. In these patients, the *CFH *TT genotype was associated with a significantly more favorable treatment response than was the *CFH *CC genotype. Specifically, AREDS supplementation was associated with a greater reduction in AMD progression (68%) in those with the low-risk TT genotype compared with those with the high-risk CC genotype (11%) [53]. No significant associations with AMD progression were seen for the *ARMS2* A69S variant.

## 4. Pharmacogenetics of PDT

Several studies have investigated the relationship between genetic variants and response to PDT (Table 2). The majority of these have focused on the AMD-associated variants *CFH* Y402H and *ARMS2* A69S. Other genes, such as those related to the angiogenesis and coagulation pathways, have also been examined.

The first AMD pharmacogenetic study involved a small series of 27 English patients treated with PDT and genotyped for *CFH* Y402H. Following treatment, patients with *CFH *CC lost a median of 12 letters of visual acuity (VA) (*P* = 0.038 compared to CFH TT), while patients with *CFH *CT lost a median of 3.5 letters (*P* = 0.087). This study suggested that patients with two *CFH* Y402H risk alleles fared worse with PDT than those with one risk allele. However, the analysis was limited by having only two treated patients with the *CFH* Y402H TT genotype, making it difficult to draw conclusions [55].

A subsequent study examined a series of 69 US patients treated with PDT and genotyped for *CFH *Y402H [56]. Adjusting for lesion type, lesion size, and pretreatment VA, the mean VA after PDT in this study was significantly worse for patients with the *CFH *TT genotype than for the *CFH *TC or *CFH *CC genotypes. This difference was significant for all patients (*P* = 0.05), as well as for the subgroup of patients with predominantly classic CNV (*P* = 0.04), but not for patients with occult CNV (*P* = 0.22). This suggests that the association between PDT outcome and *CFH* genotype in this study was driven by those patients with predominantly classic lesions. The authors examined *ARMS2* A69S genotypes as well and found no statistically significant differences among treatment outcomes with respect to genotype.

Other studies investigating PDT and *CFH* Y402H have shown no associations between this polymorphism and treatment outcome. A series of 88 Finnish patients treated with PDT was evaluated for an association with the *CFH *Y402H SNP [57]. This study used a binary responder/nonresponder outcome classification. Patients were considered to be PDT responders if the treating physician deemed the neovascular lesion to be dry without leakage on fluorescein angiography at least 12 weeks after the last treatment. PDT nonresponders were patients whose lesions did not meet this criterion. The investigators found no statistically significant differences among *CFH *Y402H genotypes with respect to PDT response or the median number of treatments required.

A study including 131 Israeli patients who were treated with PDT and genotyped for the *CFH* Y402H polymorphism used posttreatment VA as the outcome measure. In this series, there were no statistically significant differences in treatment outcomes by *CFH* Y402H genotype, with respect to initial VA, post-PDT VA, or number of PDT sessions required [58]. The same group subsequently published a series of 143 patients treated with PDT and reported that genotypes at both *ARMS2 *A69S and *HTRA1* (rs11200638) were not associated with treatment outcomes, in terms of final VA or number of PDT sessions [59].

In a series of 273 Australian patients treated with PDT and genotyped for *CFH* Y402H, participants were divided into responders and nonresponders based on posttreatment VA. Positive responders were those patients who at the final visit had either an improved or unchanged VA or those who lost fewer than 3 lines of vision (provided their final VA was better than or equal to 20/200). Negative responders were those with a final VA of worse than 20/200 or those who lost 3 or more lines of VA. In this study, there were no statistically significant differences in treatment outcomes with respect to the *CFH *Y402H genotype. Nine polymorphisms in *CRP *were also investigated in this study, and two of the nine (rs2808635 GG and rs876538 AA) were significantly correlated with more favorable response to PDT (*P* = 0.048 and *P* = 0.048, resp.) [60].

A series of 110 Japanese patients treated with PDT was screened for multiple polymorphisms in *CFH*, *HTRA1*, *VEGF*, and *PEDF*. The *HTRA1 *rs11200638 GG genotype was associated with significantly improved visual acuity outcomes and significantly less risk of recurrent disease following treatment (*P* = 0.029). The combination of two *CFH *genotypes (rs1410996 and rs2274700) was associated with a statistically significant reduction in the time interval until disease recurrence following PDT (*P* = 0.0085). In this study, there was no association between PDT response and *CFH* SNPs rs1061170 (Y402H) and rs800292, 3 *VEGF *SNPs (rs699947, rs1570360, and rs2010963), or four *PEDF *SNPs (rs12150053, rs12948385, rs9913583, and rs1136287) [61].

A series of 86 Finnish patients treated with PDT was examined in the context of three *VEGF* polymorphisms using a binary responder/nonresponder classification. As in this group's earlier study, patients were considered PDT responders if the lesion was deemed to be dry at least 12 weeks after the last treatment and PDT nonresponders failed to meet this criterion. Two *VEGF* polymorphisms (rs699947 and rs2146323) showed a statistically significant relationship to treatment, while one (rs3025033) did not. Regarding the rs699947 genotype, the C allele was associated with a significantly higher percentage of nonresponders (*P* = 0.0003). For the rs2146323 genotype, the C allele was again linked to a higher percentage of PDT nonresponders (*P* = 0.0036) [62].

Ninety patients treated with PDT for classic CNV were screened for polymorphisms in various genes affecting coagulation, including factor V G1691A, prothrombin G20210A, factor XIII-A G185T, MTHFR C677T, methionine synthase A2756G, and methionine synthase reductase A66G. Patients were classified using a binary responders/nonresponders classification. Responders were significantly associated with the prothrombin G20210A and MTHFR 677T polymorphisms. Nonresponders were significantly associated with the factor XIII-A 185T polymorphism [63]. The same group subsequently reported 84 patients treated with PDT for occult CNV that were screened for the same six coagulation factor polymorphisms. In this study, nonresponders were significantly associated with the factor XIII-A G185T mutation, and responders were significantly associated with the combination of factor V 1691A and prothrombin 20210A [64]. Of note, the MTHFR 677T polymorphism that correlated with improved outcomes in patients with classic CNV did not correlate with improved outcomes in patients with occult CNV [72].

## 5. Pharmacogenetics of Anti-VEGF Agents

A recent group of studies has reported relationships between genetic variation and response to treatment for exudative AMD with anti-VEGF agents (Table 3). At this time, all of these reports involve bevacizumab, ranibizumab, or both. 

The first study to investigate the association between genetic variants and anti-VEGF treatment for AMD was a retrospective series of 86 US patients treated with bevacizumab monotherapy. Patients were treated every six weeks until the CNV was no longer active and genotyped for the *CFH *Y402H and *ARMS2* A69S polymorphisms. The authors reported the *CFH* Y402H genotype to be significantly correlated with treatment response. Patients with the *CFH *TT genotype experienced an average VA improvement from 20/248 to 20/166; patients with the *CFH* TC genotype experienced an average VA improvement from 20/206 to 20/170; patients with the *CFH *CC genotype experienced an average VA decline from 20/206 to 20/341 (*P* = 0.016). A total of 53.7% of patients with *CFH *TT and TC genotypes gained VA with treatment, while only 10.5% of patients with the *CFH *CC genotype gained VA with treatment (*P* = 0.004). In this study, there were no statistically significant differences in treatment outcomes associated with the *ARMS2/LOC387715* genotype [65].

More recently, similar outcomes were reported by an Austrian group, which presented a prospective series of 197 patients treated with bevacizumab monotherapy. In this study, patients were also treated at six week intervals until inactivity of the lesion. Among patients studied, 41% of patients with the *CFH *CC genotype lost 3 or more lines of distance VA, as compared to 28% of patients with the *CFH *TT genotype and 26% of patients with the *CFH *TC genotype (*P* = 0.04) [66].

Four studies have investigated the pharmacogenetics of ranibizumab monotherapy for AMD. In a retrospective study of 156 US patients treated *pro re nata* with ranibizumab, the *CFH *Y402H polymorphism correlated with the number of ranibizumab injections performed. Over a 9-month period, patients with the *CFH *TT genotype required a mean of 3.3 injections; patients with the *CFH *TC genotype required a mean of 3.8 injections; patients with the *CFH *CC genotype required a mean of 3.9 injections. A recurrent event analysis demonstrated that patients with the *CFH* CC genotype were significantly more likely to require reinjections at a follow-up visit than patients with the *CFH* TT genotype (OR 1.37, 95% CI 1.01 to 1.87) [67].

A prospective series of 90 Polish patients treated with ranibizumab monotherapy was studied with respect to *CFH *Y402H and *ARMS2 *A69S. All patients experienced statistically significant improvements in VA except patients with the *ARMS2* TT genotype (two risk alleles). In addition, the *CFH *CC genotype was associated with a less significant visual acuity improvement than were the other *CFH* genotypes [68].

In an analysis of 243 eyes treated with ranibizumab monotherapy and screened for genotypes at *CFH*, *CFB*, *HTRA1*, *ARMS2*, *VEGFA*, *KDR*, *LRP5*, and *FZD4, *there was a statistically significant difference in treatment response with respect to *CFH* Y402H. In this study, two responder groups were evaluated: poor responders (≤25th percentile) and good responders (≥75th percentile). The authors reported that 38% of poor responders were associated with *CFH *CC, while only 15% of good responders were associated with *CFH* CC. Individual polymorphisms in the other genes were not significantly associated with treatment outcomes, but patients who were heterozygous at both *CFH *and *FZD4 *had significantly more favorable results; this genotype combination was identified in 36% of good responders versus 13% of poor responders [69].

A more recent series of 104 patients treated with ranibizumab monotherapy was screened for genotypes at *CFH*, *HTRA1*, and *VEGF*. There were no significant relationships between any genotype and the number of reinjections within the first 6 months. There were nonsignificant trends towards better visual acuity outcomes with certain genotypes in all 3 loci studied. The percentage of patients with a posttreatment increase in VA greater 5 letters was significantly greater among patients with the *CFH *TC genotype than those with the *CFH *TT genotype (*P* = 0.04), but there was no difference between the *CFH* CC and *CFH* TT genotypes [70].

Finally, a series of 172 patients treated with ranibizumab, bevacizumab, or a combination of the two agents was studied for polymorphisms in *APOE*. The primary endpoint was two-line improvement in visual acuity. The *APOE *ε**4 allele was associated with significantly improved treatment outcomes, as compared with the *APOE *ε**2 allele at 3-month followup (*P* = 0.02), but not at 12 months (*P* = 0.06) [71].

## 6. Pharmacogenetics of Corticosteroids

A series of 52 patients treated with IVTA for a variety of indications, including AMD, was evaluated for a relationship between IOP elevation and 6 polymorphisms in *GR* (*ER22/23EK*, *N363S*, *Bcl*I, *N766N*, and polymorphisms with introns 3 and 4) (Table 1). There were no statistically significant associations between any individual polymorphism, or by haplotype analysis, with IOP elevation following treatment with IVTA [54].

## 7. Summary

Several pilot pharmacogenetic studies have reported some evidence of genotype-phenotype interactions with respect to treatment outcomes using AREDS vitamins, PDT, ranibizumab, and bevacizumab. At this point, the data are conflicting and no definite conclusions may be drawn. The results may be inconsistent because of underlying differences in baseline genetic characteristics, differences in underlying CNV lesion characteristics (classic versus occult, chronicity, etc.), differences in study endpoints (visual acuity, anatomic response, number of retreatments required, etc.) statistical analysis (use of continuous outcome versus dichotomizing these variables), or other factors. 

At this time, pharmacogenetics remains a research tool rather than an option for daily clinical use. Nevertheless, there appears to be a relationship between *CFH*, *ARMS2*, and perhaps other genes with respect to treatment outcomes. As we continue to collect data from clinical trials, these relationships may become more apparent.

## Figures and Tables

**Table 1:** Pharmacogenetics of AREDS vitamins and intravitreal triamcinolone acetonide.

Treatment	Genes	Result
AREDS vitamins	*CFH* and *ARMS2 *	*CFH *TT associated with greater reduction in disease progression; no effect with *ARMS2* [53]
IVTA	Multiple	No association between IOP elevation and any gene [54]

AREDS: Age-Related Eye Disease Study; IOP: intraocular pressure; IVTA: intravitreal triamcinolone acetonide.

**Table 2:** Pharmacogenetics of photodynamic therapy.

Treatment	Genes	Result
PDT	*CFH*	*CFH* CC associated with worse visual outcomes [55]
PDT	*CFH *and *ARMS2 *	*CFH* TT associated with worse visual outcomes, especially in predominantly classic CNV; no association with *ARMS2* [56]
PDT	*CFH*	No association with PDT responders versus nonresponders [57]
PDT	*CFH*	No association with visual acuity outcomes [58]
PDT	*ARMS2 *and *HTRA1 *	No association with visual acuity outcomes or number of PDT sessions with either gene [59]
PDT	*CFH *and* CRP *	No effect with *CFH*; 2 of 9 *CRP* polymorphisms associated with more favorable response to treatment [60]
PDT	*CFH*, *HTRA1*,* VEGF*, and* PEDF *	*HTRA1* GG associated with more favorable treatment outcomes; combination of 2 *CFH* genotypes associated with reduced time interval until disease recurrence; no association with other genes [61]
PDT	*VEGF*	2 polymorphisms associated with response to treatment [62]
PDT	Multiple	In classic CNV, prothrombin and MTFHR associated with PDT responders; factor XIII-A associated with PDT nonresponders; factor V, methionine synthase; methionine synthase reductase not associated with PDT response [63]
		In occult CNV, combination of factor V and prothrombin associated with PDT responders; factor XIII-A associated with PDT nonresponders; MTFHR, methionine synthase; methionine synthase reductase not associated with PDT response [64]

CNV: choroidal neovascularization, PDT: photodynamic therapy.

**Table 3:** Pharmacogenetics of antivascular endothelial growth factor therapy.

Treatment	Genes	Result
Bevacizumab	*CFH *and *ARMS2 *	*CFH *CC associated with worse visual outcomes; no association with *ARMS2 *[65]
Bevacizumab	*CFH*	*CFH* CC associated with worse visual outcomes [66]
Ranibizumab	*CFH*	*CFH *CC associated with more injections performed [67]
Ranibizumab	*CFH *and *ARMS2 *	*ARMS2* TT associated with worse visual outcomes; *CFH *CC associated with relatively worse visual outcomes [68]
Ranibizumab	Multiple	*CFH* CC associated with poor treatment response; combination heterozygotes at *CFH* and *FZD4 *associated with more favorable outcomes; no association with *CFB*, *HTRA1*, *ARMS2*, *VEGFA*, *KDR*, and *LRP5 *[69]
Ranibizumab	*CFH*, *HTRA1*, and *VEGF *	*CFH* TC associated with better visual outcomes; no association with number of injections with any gene [70]
Bevacizumab and/or ranibizumab	*APOE*	*APOE ε*4 associated with better treatment outcomes [71]
